# Representing dispositions

**DOI:** 10.1186/2041-1480-2-S4-S4

**Published:** 2011-08-09

**Authors:** Johannes Röhl, Ludger Jansen

**Affiliations:** 1 Department of Philosophy, University of Rostock, August-Bebel-Straße 28, 18051 Rostock, Germany

## Abstract

Dispositions and tendencies feature significantly in the biomedical domain and therefore in representations of knowledge of that domain. They are not only important for specific applications like an infectious disease ontology, but also as part of a general strategy for modelling knowledge about molecular interactions. But the task of representing dispositions in some formal ontological systems is fraught with several problems, which are partly due to the fact that Description Logics can only deal well with binary relations. The paper will discuss some of the results of the philosophical debate about dispositions, in order to see whether the formal relations needed to represent dispositions can be broken down to binary relations. Finally, we will discuss problems arising from the possibility of the absence of realizations, of multi-track or multi-trigger dispositions and offer suggestions on how to deal with them.

## Background

Terms for dispositions and their cognates like tendencies or propensities are important in biomedical data structures [1]. SNOMED CT, for example, contains *Determination_of_disposition_of_blood_product* (*procedure*) as a procedure, *Character_trait_finding_of_sadistic_tendency* (*finding*) as a clinical finding, *Propensity_to_adverse_reaction_to_drug* as a disorder (January 2010 Release) [2]. Dispositions relevant for the biomedical domain comprise also the tendency of a patient for nausea, the capacity of drugs like aspirin to relieve pain, and the dispositions of molecules to undergo certain chemical reactions under certain circumstances. Such dispositions are important, because in the medical domain we are in general not only interested in what actually happens, but also in what could happen, given the causal properties of the patient, as we may need this knowledge, e.g., in order to prevent something from happening by taking precautions, and disposition ascriptions entail which occurrences could take place if certain conditions were met.

### Dispositions – what and why

A disposition is a causal property that is linked to a realization, i.e. to a specific behaviour which the individual that bears the disposition will show under certain circumstances or as response to a certain stimulus (trigger). Common examples are fragility (the disposition to break when dropped) or solubility (the disposition to dissolve when put in water). There has been a lively and controversial philosophical debate on the status and nature of dispositions in the last decades. Although there have been attempts to eliminate dispositional concepts from scientific discourse (Carnap [3], Quine [[4], pp. 8-11]) or to “reduce” dispositions to some other entities, usually non-dispositional properties (Armstrong [[5], pp. 70-80]), these attempts are considered to be failures and a realist position has been defended by recent authors (Mumford [6], Ellis [7,8], Molnar [9]) that takes dispositions seriously as a kind of properties in their own right. We adopt here such a realist position with respect to dispositions that can not only be supported by philosophical arguments, but is also close to a pre-philosophical understanding and adequate to scientific practice. Two aspects are central characteristics of dispositions [10,6,9]: On the one hand, disposition ascriptions are ascriptions of properties like fragility or solubility to their bearers – properties that are causally relevant for processes that involve the disposition’s bearer as a participant. Accordingly, dispositions are a subtype of properties. On the other hand, disposition ascriptions entail counterfactual conditionals. That a material entity x is the bearer of an instance d of a disposition type *D* implies that if x was under certain circumstances c of type *C*, x would participate in a realization process r of type *R*, the realization of the disposition. This counterfactual entailment of a disposition ascription expresses the relation of the disposition to a possible realization; one can say that dispositions "point beyond themselves" - namely to their realizations. We will briefly discuss two applications of dispositions in the ontology of biology and medicine that show why these features make dispositions crucial in many respects.

### Applications (1): dispositions and interactions

A general feature of dispositions is that they can provide a connection between two major types of entities we recognize in our ontology: independent continuants and occurrents. [Basic Formal Ontology (BFO), cf. [11] Independent continuant entities are what we would think of as “things”, i.e., entities that can change in time, but have no temporal parts. If a patient gains 10 kg, he changes in that respect, but his identity is not affected by that change. The heavier patient is the same person as before, not a later temporal part of a four-dimensional entity. On the contrary, occurrents are entities like events or processes that take place in time and have temporal parts (phases or stages): The second half of the final match of the soccer world cup is a temporal part of that final match. Independent continuants are the bearers of the dispositions, because dispositions are a subtype of properties and as such always need bearers. The realizations of dispositions are processes that have the bearers of the dispositions as participants.

When describing complex interactions, e.g. between types of molecules, one option is to model interactions as direct relations with the interaction partners as relata. This leads to relations of higher arity, depending on the number of interaction partners and also to a multiplication of relations, because every interaction type needs its own relation. As it is widely recommended to restrict oneself to a small set of basic binary relations [12], Schulz and Jansen [13] suggest the following reification strategy: As a first step, interaction processes are taken as occurrents, entities in their own right, to reduce the arity. The interaction process, then, stands in the binary relation, *has_participant*, to two or more continuants. Dispositions for the respective interactions can then be ascribed to the potential participants of the interactions. In this fashion one can analyze complex interactions in terms of interaction processes and independent continuants, the participants of these processes, and their dispositions in virtue of which they enter into the interactions. This approach is in line with prominent positions in the recent philosophical debates on causality and laws of nature that take interaction processes as realizations of the dispositions of the participants in interactions. Thus, dispositions connect the static structure of the world, i.e. the natural kinds of continuants, with the dynamical structure, i.e. the types of possible and actual causal processes [7,8].

### Applications (2): diseases as dispositions

The central role of dispositions is shown by the fact that some recent biomedical ontologies define diseases as dispositions towards pathological processes (cf. [14,15], and OGMS, the Ontology for General Medical Science, [16]). In OGMS a disease is characterized by three categorically distinct aspects *disposition*, *disorder* and *course of disease*. First, there is the disease as a *disposition* that can (but need not) cause various symptoms. The corresponding *disorder* is the basis of the disposition, i.e. the structure of properties that bring the disposition in question with it. The *course of disease* is the realization of the disease as a disposition; it is an occurrent or process. (Although this way of modelling diseases seems to be useful to us, this is not the place to defend it against alternative disease models. In the present context, it serves mainly as an example for the use of dispositions in present medical ontologies.)

The usefulness of this distinction between the disposition for and the realization of the course of disease becomes apparent when we think of drugs as “antidotes” that counteract or block the disposition that would usually be realized as the disease course [14]. Two things should be noted: First, the disorder is specific for the disease. Not any presence of infectious agents in some organ means that the patient has a particular disease, but it must be the type of agents in the specific place for the disease in question. Second, this model implies that the term “disease” without further qualification is in fact ambiguous: The disorder and its associated disposition may in some cases be “dormant” until it is realized in the disease course (and it may even never be realized in some patients). This distinction is important, because it helps to describe the case of patients having the disease qua disorder or disposition but no symptoms, because the realization is blocked by an additional factor (e.g., by medication that counteracts the disease). A patient may carry the infectious agents and even spread them without exhibiting any typical symptoms, as it is often the case with infectious mononucleosis (Pfeiffer's disease) and other infectious diseases.

In this context it is also important that the relation between a disposition and its bearer is not confounded with the relation between a disposition and its basis, as bearer and base belong to different top level categories. Using BFO categories as our top level ontology [11,17], we can align them as follows: The bearer is an independent continuant or Aristotelian substance, i.e. the diseased organism or a part thereof. The disorder is a property (or a set of properties) of the bearer, thus it is a dependent continuant. We will come back to that distinction later (in the section “Base_of”).

**Figure 1:**
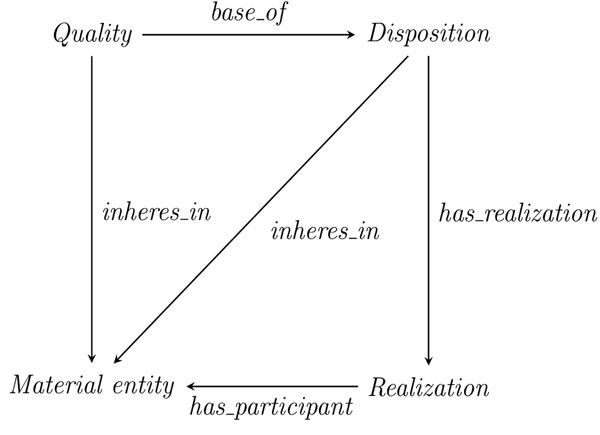
Fundamental relations of dispositions

Here we argue that in order to clarify the dependencies among bearer, base and realization of a disposition, the OGMS-“triangle” should be extended to a “square” including the substantive bearer, the base, the disposition and the realization as its corners. We shall now further inquire into the edges of this “square” (cf. Figure 1), i.e. the formal ontological relations between these (types of) entities.

## Methods

### Formal relations for the representation of dispositions

In the top-level ontology BFO (“Basic Formal Ontology”), dispositions are taken as a type of “realizable entity” (with two siblings, “function” and “role”) which in turn is a subclass of “dependent continuant” (with sibling “quality”)[18]. Thus there are two relations that are in any case necessary for a disposition: As a dependent continuant it needs an independent continuant as its bearer. And as a realizable it points to a realization, a process entity that will be realized, given the appropriate conditions. But disposition ascriptions can be considerably more complex: A disposition ascription according to the schema “x has disposition D for realization R with trigger T under circumstances C with probability P” as suggested in [13] has no less than six relata: the independent continuant x that has the disposition, the disposition D, the realization R (process), the trigger T and the circumstances C, and a probability P of the realization. The Description Logics that are used by ontology languages like OWL, however, can deal well with binary relations only [19,20].

In the following, we will try to reduce this complexity in a systematic fashion, using binary relations only. However, considerable difficulties will remain. And clearly, the triple <trigger, disposition, realization> is the core of the usual conception of dispositions, so it seems a rather contrived move to split this ternary relation up. Thus the following should be considered as a compromise between what we see as the essential ontological connections and the restrictions forced by the practical necessity of using only binary relations. It should also be noted that the results presented in this paper are to be understood as the theoretical basis for the use of dispositions in domain ontologies. Their performance in concrete applications has to be evaluated in the future.

### Expanding expressibility

In the examples from SNOMED CT mentioned in the introduction, dispositions are only characterized by means of their realizations. Similarly, in the OGMS approach to diseases, diseases as dispositions are only related to the bodily disorder underlying it and the pathological process. This makes things easy when it comes to formal relations, for only few binary relations are required to represent the OGMS triangle [14]. The approaches of SNOMED CT and OGMS, however, have a rather restricted expressivity. For they cannot distinguish between dispositions for the same realization albeit under several circumstances. But then disposition ascriptions seem to become trivial. For anything, or so the stock argument goes, is fragile under some circumstances. A rose petal is usually quite flexible, but when it is frozen, it will break when twisted. And virtually any human being has a tendency to nausea, if the circumstances are sufficiently extreme. The meaning intended by the developers of SNOMED CT is, of course, that some patients have a tendency to develop nausea in circumstances in which healthy people do not exhibit this tendency. A typical move in this situation is to refer to standard conditions for the realization. For some parameters, there are conventions about which range of the parameters corresponds to standard conditions, but in other cases it is not clear which conditions are standard. In any case, this move will deprive us of the possibility to say anything about reactions in special conditions. In order to be able to make such points, we have to relate dispositions not only to their realizations, but also to the events or processes that trigger these realizations.

It is true that in many cases the trigger of a disposition might not be known, and in other cases we may even have good reasons to assume that no trigger is involved. Current nuclear physics, for example, argues that the dispositions responsible for the decay of Uranium nuclei do not have any trigger but are irreducibly probabilistic. Nevertheless, we need to take triggers into account for many biomedical cases. Otherwise we cannot distinguish, say, the pathological tendency to bleed that comes along with haemophilia from the physiological tendency to bleed when, e.g., a child looses a milk tooth.

Moreover, some medical dispositions are mainly classified by the triggers, as, e.g., hypersensitivities or allergies [21]. Obviously, without taking triggers into account, an ontology of dispositions cannot include this class of medically important dispositions. In such cases, however, often the allergenic substances are said to be the the triggers of the allergic reaction, and substances are not events. But we can easily accommodate this, for it is not the substance as such that is the trigger of the allergic reaction, but the contact between the patient and the allergen, or the patient's being exposed to the allergenic substance. And these are, again, events.

### Starting with the simple

In order to get started, we will first consider simplified types of disposition ascriptions at the expense of more complicated examples (cf. Table 1). We will (1) concentrate on “sure fire dispositions”, i.e. such dispositions that will necessarily be realized given the respective realization conditions. Hence we will abstract from all those dispositions that even in “ideal conditions” will be realized with a certain probability only, and which are often called “propensities” [22] or “tendencies” [1]. This seems to be ontologically sound, as the ascription of a probability does not establish any ontological addition. For the purpose at hand, a probability is a real number from the interval ]0,1[, and the ascription of the probability is a function of the tuple <disposition, realization> into that interval. It seems plausible that such a function can be attached after we have disentangled the main complex. We will also (2) ignore the distinction between “trigger” and “background conditions” and focus on triggering processes only. The ontological basis for this distinction is that triggers come along with changes (the striking of the match), while background conditions may remain constant (presence of oxygen). But to a large degree, this distinction is a pragmatic one. Usually the background conditions are taken for granted, while the trigger is thought of as the causal factor that (a) makes a difference and (b) can be or actually is influenced by human action. But both the trigger and the background conditions are necessary conditions for the realization. In this paper, we focus on the trigger, because background conditions can comprise categorially diverse kinds of entities (location, presence of oxygen, milieu of certain pH value, concentration) and it is currently not clear at all how many of these conditions are to be analyzed ontologically. It is very much desirable to integrate such conditions, once their ontological status becomes clear, because, e.g., the realization of a disease may be influenced by such additional conditions, be they health conditions of the patient or conditions induced by a drug treatment. Moreover, we will (3) start with cases where one type of disposition is correlated with exactly one type of realizations, i.e. we exclude dispositions that allow for several types of realizations (so-called “multi-track dispositions”). Finally, we will (4) consider only cases which involve exactly one type of trigger, i.e. we exclude all dispositions that can be triggered by processes of more than one type, as well as all those dispositions that need no trigger at all. These are severe restrictions, but it should be noted that there are many cases of everyday dispositions that seem to conform to them reasonably well, e.g. the striking of a match and its subsequent inflammation can be considered to be the unique trigger and realization of the match's inflammability disposition. A biochemical example is the disposition of a sample of Gram-positive bacteria like *Staphylococcus aureus* to turn dark blue in the process of Gram-staining. Medical examples include the disposition for the patellar (“knee jerk”) reflex triggered by striking the spot below the patella, and the tendency to bleed without proper blood coagulation of a haemophiliac patient triggered by a lesion or the allergic reaction of a patient hypersensitive to penicillin. Moreover, we (5) ignore all questions regarding non-monadic dispositions for the purpose of this paper.

**Table 1:** Types of surefire dispositions

	… for exactly one type of realization		… for more than one type of realization	
	
	… with exactly one trigger-type	… with more than one possible trigger-type	… with exactly one trigger-type	… with more than one possible trigger-type
Exactly one type of disposition …	Single track		Multi-track	

More than one type of disposition …	“Polycausality”		“Chaos”	

## Results

### Binary formal relations

We now characterize the basic binary formal relations for the representation of dispositions. We start with the level of particulars. We follow the OBO Relation Ontology [12] and regard as primitive the particular level relations **instance_of** (which holds between a particular and its classes, both for processes and continuants) and **has_participant** (which holds between particular processes and particular continuants). We also adopt the convention of using boldface for relations involving particulars and italics for relations between types. We use lower case letters for variables for instances and italicized upper case for variables for classes, while we both italicize and capitalize terms for classes. In addition, we use the particular level relation **inheres_in**, which holds between individual instances of dependent and independent continuants [13]. It expresses a kind of one-sided ontological dependence, i.e. if p **inheres_in** x, then it is possible that x exists without p, but not that p exists without x.

#### Has_disposition

Given these primitive relations, we can define a relation **has_disposition** along the following line:

x **has_disposition** d :=Ǝ*D d*** instance_of*** D* ⋀ *D is_a Disposition* ⋀ d **inheres_in** x (1)

With the help of these particular level relations we can now start to define the wanted universal level relation *has disposition* (which is a subrelation of *has_property* as defined in [13]). This relation will be needed to express that certain types of things have some dispositions essentially. In these cases, *all* instances of a certain kind will have instances of the disposition type inhering in them, e.g. all aspirin pills have the disposition to relieve pain, and all sugar has the disposition to dissolve in water:

*A has_disposition D* := ∀x x **instance_of*** A* → Ǝy y **instance_of*** D* ⋀ x **has_disposition** y (2)

We have, however, to be careful when applying this relation, as in many cases only some instances of a given type will have a certain disposition. E.g., only some patients have the disposition to show an allergic reaction to penicillin, and only some mosquitoes have the disposition to transmit malaria.

#### Inheres_in

The inverse relation of **has_disposition** is a sub-relation of the general **inheres_in** relation. Like all property instances, disposition instances need to inhere in some bearer in order to exist, as dispositions are dependent continuants. Even if particular dispositions are of the same type, their bearers do not have to be of the same type. E.g., tablets of quite different types, like aspirins and paracetamols, can be bearers of instances the same disposition type to relieve pain e.g. aspirin as well as paracetamol. If, however, all instances of a disposition have bearers of the same type, we can represent this by means of the *inheres in* relation, which we can define as follows:

*D inheres in B* := ∀x (x **instance_of*** D* → Ǝy y **instance_of*** B* ⋀ x **inheres_in** y (3)

#### Has_realization

In addition, it will be useful to have relations linking a disposition to its realization and its trigger. It is, however, not possible to define such relations according to the usual all-some semantics, because the whole point of disposition ascriptions is that not every instance of a type of disposition needs to be realized: “For all instances x of *D* there exists an instance r of the process type *R*, such that r is the realization of x” is normally wrong - otherwise, all dispositions would always be realized. But a particular disposition of a particular material entity may never be realized and there are even cases in which *no* token of a disposition type is ever realized, because the triggering circumstances are never met. This is a feature, not a bug, as we want dispositions to be real without actual realizations. It is possible that there is a type of disposition of which no instance will ever be realized, like, the disposition of a nuclear power plant to explode, the realization of which is prevented by highly sophisticated technical machinery. (In most biomedical cases, however, usually some tokens of the disposition will be realized or will have been realized before.) We use **has_realization** as the primitive relation that connects a disposition instance with any process instance which is its realization. This is both an ontological and a causal connection, and we can roughly characterize it by saying that the realization has been brought about by the disposition. Instead of using the all-some apparatus, we introduce *has_realization* by setting up a set of value restrictions according to the following scheme: A type of process R is the realization type of a disposition type D if and only if any instance of D is realized, then the realization is of type R; i.e.

*D has_realization R* := ∀x (x **instance_of*** D* → (∀y (x **has_realization** y → y **instance_of*** R*) (4)

#### Has_trigger

We will introduce two distinct relations to describe the trigger of a disposition. The trigger relation can have these two variants as we can split the fundamental nexus <disposition, trigger, realization> in two ways. When focusing on the disposition we consider the relation between the triggering process and the disposition that is realized because of the trigger, e.g. between the fragility of a glass and its dropping. On the other hand it seems natural to think of a (causal) connection holding between the triggering process and the realization of the disposition (the dropping of the glass triggers its breaking). Therefore we introduce a relation **has_trigger_D_** that holds between the disposition and the triggering process and a relation **has_trigger_R_** that holds between the realization process and the triggering process. Now some dispositions (e.g., the disposition causing the patellar reflex) may be triggered several times, evoking as many realizations (knee jerks). On the other hand, any realization instance of a single-trigger disposition instance has, by definition, exactly one trigger instance. For this reason, we take the relation **has_trigger_R_** as basic and use it to define the relation **has_trigger_D_** as follows:

d **has_trigger_D_** t ⇔ Ǝr (d **has_realization** r ⋀ r **has_trigger_R_** t) (5)

Note that the relations with bold names concern the instance level relations. Given our simplifying assumption that there is exactly one type of realization and exactly one type of trigger for each disposition, we can formulate the corresponding relations on the level of universals. But the same cautionary remarks as in the case of *has realization* apply: Not for every particular disposition there is a particular trigger process, but at most for those particular dispositions that become actually realized. Thus, again, we cannot use the all-some apparatus but must express our knowledge about types of trigger processes using value restrictions along the following lines:

*D has_trigger_D_ T* := ∀x (x **instance_of*** D* → ∀y (x **has_trigger_D_** y → y **instance_of*** T*)) (6)

If a disposition requires exactly one type of trigger, then it holds for *has trigger_R_* that every realization must have been triggered by an instance of that class and we can define:

*R has_trigger_R_ T* := ∀x (x **instance_of*** R* → Ǝy y **instance_of*** T* ⋀ x **has_trigger_R_** y) (7)

#### Base_of

Important discoveries about dispositions regard the question how dispositions are brought about through the interplay of micro-level structures and their properties. Such a microstructure is normally labelled as the “base” of a disposition. For example, the solubility of salt is based on the molecular structure of NaCl that allows the polarized water-molecules to break the ion bonds. While there are good reasons to assume that there are dispositions on the quantum physical level that do not have a non-dispositional “grounding”, dispositions in the biomedical domain can probably always be connected to a set of properties that account for their existence. We do not introduce a further primitive relation on the particular level, but characterize the universal level relation through the need of a disposition instance inhering in the bearer of the base quality:

(*Q is_a Quality* ⋀ *D is_a Disposition* ⋀ *Q base of D*) **→** ∀q (q **instance_of*** Q*** →** Ǝb (q **inheres_in** b ⋀ Ǝd (d **instance_of*** D* ⋀ d **inheres_in** b))) (8)

While equal bases bring along instances of the same type of dispositions, the inverse does not hold. As a rule, in the biomedical domain instances of the same type of disposition can be constituted by instances of different types of base qualities. Our general formulation leaves leeway for several candidates for bases. If a patient lacks a certain enzyme, one could either take that absence as the basis of the disposition for the resulting pathophysiological processes. Or, to avoid “negative entities” like absences, one could say that the whole pathological (micro-)structure of an organ is the base of the respective disposition for a pathological process. Such a pathological structure can be further analyzed into its constituents and their respective dispositions. Thus we get something like a “cascade” of dispositions: The disposition to produce insufficient amounts of the enzyme leads to the bodily state characterized by lack of the enzyme and that state is in turn the base for the disposition for pathological processes resulting from that state. An advantage of disposition talk is, however, that we do not have to name or even to know the base. E.g., we often know the dispositions of a drug from clinical studies without knowing anything about the molecular mechanism that is the base of these dispositions.

This approach seems more general and more appropriate than the suggestion implied in OGMS [14] to take a material part of the bearer of the disposition as the “base” of that disposition. In fact, the latter approach seems to be based on a conflation of the distinct roles of bearer and base of a disposition. There are some special cases where this latter approach works to some extent, as the disposition of the whole is grounded in a respective disposition of a proper part of the whole. A patient's disposition to deal with certain chemicals may be based on the respective disposition of his liver, one of the proper parts of this patient. But generally, as the above example of the water-solubility of NaCl shows, this disposition is not simply based on the fact that this substance has Na^+^ ions as parts, but on the properties of the micro-constituents and their specific structure, hence on a set of qualities of the whole soluble stuff. And of course, the liver's disposition to remove harmful substances from the rest of the body is based on the functional structure of that organ as a whole. A whole x may have a disposition d, because one of its parts p is the bearer of this disposition. But then we are still faced with the question what confers the disposition d on that part p. In any case, the relation between a disposition and its bearer is ontologically different from the relation between a disposition and its base. The former is a relation between a disposition (a dependent continuant) and an independent continuant and the latter is a relation between a disposition and a structured aggregate of dependent continuants.

### Principles for surefire dispositions

To connect these relations with each other we now suggest a number of postulates that are intuitively plausible for surefire dispositions. These principles could be added as axioms to respective knowledge bases. The first one is the *realization principle*, i.e. the principle that if a disposition and its trigger are given, the realization will happen:

(Ǝ*D D is_a Disposition* ⋀ Ǝ*T T is_a Process* ⋀ Ǝ*R R is_a Process* ⋀ *D has_trigger_D_ T* ⋀ *D has_realization R* ⋀ Ǝd d **instance_of*** D* ⋀ Ǝt t **instance_of*** T*) → Ǝr r **instance_of*** R* (9)

As before, we suppress all time indices in the present paper for the sake of brevity. The second principle is the bearer principle which is valid for all kinds of dispositions, i.e. the principle that the bearer of the disposition is a participant of the respective realization process. The following formulation of this principle, however, relies on the realization principle, and is thus only valid for surefire dispositions:

(Ǝ*D D is_a Disposition* ⋀ Ǝ*T T is_a Process* ⋀ Ǝ*R R is_a Process* ⋀ *D has_trigger_D_ T* ⋀ *D has_realization R* ⋀ Ǝd d **instance_of*** D* ⋀ Ǝt t **instance_of*** T* ⋀ Ǝb d **inheres_in** b) → Ǝr (r **instance_of*** R* ⋀ r **has_participant** b) (10)

It seems plausible that a process normally is the realization of several different dispositions of the participants of the process. Therefore the converse of (10) will not hold; we cannot infer uniquely from an occurring process which dispositions it is a realization of. Some authors even speak of “complementary dispositions” that come in pairs [14,23]: For example, the disposition of a type of key to open a particular type of lock is complementary to the disposition of that type of lock to unlatch when that kind of key is inserted and turned. The disposition of the key can only be realized, if the disposition of the lock is being realized, too. In molecular biology, the key-lock-metaphor is often used for the dispositions of molecules for reaction that are to some extent grounded in the molecule's geometry [14]. According to this view, a movement is the realization of both an active disposition to cause motion and a passive disposition to undergo motion. But any participant participates in the respective process in virtue of one of its passive or active dispositions.

Finally, we should remark that the following relations hold between the top-level categories in our square diagram discussed above (Figure 1):

Disposition inheres_in Material Entity

Quality inheres_in Material Entity

Disposition has_realization Process

Realization has_participant Material Entity

Quality base_of Disposition

Disposition has_base Quality

As we have already noted before, the last two assertions might not be true in unrestricted generality, whereas they seem to be plausible for all biomedical entities.

## Discussion

We characterized the main ontological dependencies and relations between dispositions, their bearers, triggers and realizations. Starting with several simplifying assumptions, these originally complex relations were split up in binary relations and formally defined in first order logics. The simple cases that can be modelled in this fashion seem rather limited, and there would be considerable gains in expressibility, if at least ternary relations could be used. We will sketch an approach to extend our basic analysis to include one set of more interesting and realistic cases: multi-track and multi-trigger dispositions. A fragile thing can break into pieces, but it can also crack or splinter – and all of those would count as realizations of fragility. We can also trigger the fragility disposition in different ways, like striking, throwing onto a hard surface etc. Similar things appear in medical cases, in which the realization of a disease, the course of the disease leading to certain symptoms, may cover a wide spectrum of behaviour.

There are several options here. One would be to analyze such dispositions by “fine-graining”, i.e. “splitting up” a multi-track or multi-trigger disposition into a set of more precisely specified ones, each with its specific triggering conditions and realization. To these finely grained dispositions our analysis could be applied, because they obey the single-track/single-trigger restrictions. In some cases there are systematic connections between the fine-grained dispositions as, for example, in the ability of a litmus paper to exhibit different shades of blue or red if put into a solution depending on the pH value of that solution. Here we have fine-grained dispositions that are systematically unified, because they are all shades of colour and all their triggers are contacts with solutions of pH values. But this strategy does not always seem appropriate to some central applications in the biomedical domain. A disease may have different disease courses as its realizations, but still be the same disease, and because the underlying disorder is the same, it cannot be split up in fine-grained dispositions. Hence, we would lose one of the advantages gained by introducing dispositions into an ontology in the first place.

Another option would then be to give a disjunctive list of the possible realizations of multi-track dispositions. In many cases, these realizations will exclude each other, but in others they will not be mutually disjoint: The realization of a particular disease may involve fever *or* nausea *or* both of these. For the medical domain this approach seems to be the most appropriate. More formally, if we give up the single realization condition, the multitrack disposition *D* has now several possible realizations types *R*_1_, *R*_2_…, *R*_n_. Accordingly, we can set up a defined class R* as the union class of all these realization types:

x **instance_of** R* ⇔ x **instance_of*** R_1_* ∨ x **instance_of*** R_2_* ∨…∨ x **instance_of*** R_n_* (11)

Having this defined class at our disposal, we can state (12) as a modified version of (4):

D has_realization R* := ∀x (x **instance_of*** D* → (∀y (x **has_realization** y → y **instance_of** R*) (12)

With the help of R* we can also formulate (13) as a modification of the realization principle (9) for multitrack dispositions:

(Ǝ*D D is_a Disposition* ⋀ Ǝ*T T is_a Process* ⋀ Ǝ*R_1…n_ R_i_ is_a Process* ⋀ ƎR* (∀x (x **instance_of** R* ⇔ x **instance_of*** R_1_* ∨ x **instance_of*** R_2_* ∨…∨ x **instance_of*** R_n_*) ⋀ *D has_trigger_D_ T* ⋀ D has_realisation R* ⋀ Ǝd d **instance_of*** D* ⋀ Ǝt t **instance_of*** T*)) → Ǝr (r **instance_of** R*) (13)

One can proceed similarly for multi-trigger dispositions by modifying definition (7), replacing “*T*” by “T*”, with T* being defined as the union class of all alternative trigger types *T*_1_, *T_2_*…, *T*_n_ of *D:*

*x*** instance_of** T* ⇔ *x*** instance_of*** T_1_* ∨ x **instance_of*** T_2_* ∨…∨ x **instance_of*** T_n_* (14)

These replacements look almost trivial, but it should be noted that the defined union classes R* and T* are far less informative than the original types of processes that were assumed to be “natural” types, not disjunctively defined ones. In future work we will investigate if and how these options can be related to the formal relations presented above and inquire into their potential to structure biomedical ontologies of various sizes.

## Conclusions

For the restricted set of single-trigger and sure-fire dispositions the essential ontological relations of dispositions to their bearers, triggers and realization processes can be modelled, using only binary relations with logically rigorous definitions. However, extending this approach to more complex cases seems to be difficult. Some options how to proceed have been sketched and should be explored further in future work.

## Competing interests

The authors declare that they have no competing interests.

## Authors' contributions

Both authors contributed equally to the present work.
